# IL-1R1-Dependent Signals Improve Control of Cytosolic Virulent Mycobacteria *In Vivo*

**DOI:** 10.1128/mSphere.00153-21

**Published:** 2021-05-05

**Authors:** Sanne van der Niet, Maaike van Zon, Karin de Punder, Anita Grootemaat, Sofie Rutten, Simone J. C. F. M. Moorlag, Diane Houben, Astrid M. van der Sar, Wilbert Bitter, Roland Brosch, Rogelio Hernandez Pando, Maria T. Pena, Peter J. Peters, Eric A. Reits, Katrin D. Mayer-Barber, Nicole N. van der Wel

**Affiliations:** a Electron Microscopy Centre Amsterdam, Amsterdam University Medical Centre AMC, Amsterdam, The Netherlands; b Netherlands Cancer Institute, Amsterdam, The Netherlands; c Amsterdam University Medical Centre VUMC, Amsterdam, The Netherlands; d Unit for Integrated Mycobacterial Pathogenomics, CNRS UMR 3525, Paris, France; e National Institute of Medical Sciences and Nutrition, Mexico City, Mexico; f Department of Health and Human Services, Health Resources and Services Administration, Healthcare Systems Bureau, National Hansen’s Disease Programs, Baton Rouge, Louisiana, USA; g Inflammation and Innate Immunity Unit, Laboratory of Clinical Immunology and Microbiology, National Institute of Allergy and Infectious Diseases, National Institutes of Health, Bethesda, Maryland, USA; University of Kentucky

**Keywords:** *Mycobacterium tuberculosis*, *Mycobacterium leprae*, *Mycobacterium marinum*, cytosolic localization, IL-1 receptor 1, phagosome, lysosome, phagolysosomal fusion, lysosomes, phagosomes

## Abstract

Mycobacterium tuberculosis infections claim more than a million lives each year, and better treatments or vaccines are required. A crucial pathogenicity factor is translocation from phagolysosomes to the cytosol upon phagocytosis by macrophages. Translocation from the phagolysosome to the cytosol is an ESX-1-dependent process, as previously shown *in vitro*. Here, we show that *in vivo*, mycobacteria also translocate to the cytosol but mainly when host immunity is compromised. We observed only low numbers of cytosolic bacilli in mice, armadillos, zebrafish, and patient material infected with M. tuberculosis, M. marinum, or M. leprae. In contrast, when innate or adaptive immunity was compromised, as in severe combined immunodeficiency (SCID) or interleukin-1 receptor 1 (IL-1R1)-deficient mice, significant numbers of cytosolic M. tuberculosis bacilli were detected in the lungs of infected mice. Taken together, *in vivo*, translocation to the cytosol of M. tuberculosis is controlled by adaptive immune responses as well as IL-1R1-mediated signals.

**IMPORTANCE** For decades, Mycobacterium tuberculosis has been one of the deadliest pathogens known. Despite infecting approximately one-third of the human population, no effective treatment or vaccine is available. A crucial pathogenicity factor is subcellular localization, as M. tuberculosis can translocate from phagolysosome to the cytosol in macrophages. The situation *in vivo* is more complicated. In this study, we establish that high-level cytosolic escape of mycobacteria can indeed occur *in vivo* but mainly when host resistance is compromised. The IL-1 pathway is crucial for the control of the number of cytosolic mycobacteria. The establishment that immune signals result in the clearance of cells containing cytosolic mycobacteria connects two important fields, cell biology and immunology, which is vital for the understanding of the pathology of M. tuberculosis.

## Abstract (precis)

For decades, Mycobacterium tuberculosis has been one of the deadliest pathogens known. Despite infecting approximately one-third of the human population, no effective treatment or vaccine is available.

## INTRODUCTION

Mycobacterium tuberculosis not only is one of the deadliest pathogens in history but also continues to claim an estimated 1.5 million human lives per year (1) and is a big threat for the future as multidrug-resistant strains are arising, with no effective treatment or vaccine available. The treatment success rate is just 56%, and in approximately 6.2% of cases, infection is caused by extensively drug-resistant *M. tuberculosis* (1). Furthermore, *M. tuberculosis* is the leading cause of death among HIV-infected patients. In patients with AIDS, tuberculosis (TB) can thrive because their immune system is impaired by CD4^+^ T cell loss, which secondarily affects many other immune compartments (reviewed in reference 2).

For an effective response to *M. tuberculosis* infections, both the innate and the adaptive immune systems are important. The development of an active pulmonary *M. tuberculosis* infection is related to a disordered immune balance, which results in the inability of the host to keep the infection under control (3). The first immune response is the innate response (reviewed in reference 4), including a series of cells that come into contact with *M. tuberculosis*, such as alveolar macrophages. Alveolar macrophages provide a nutritionally permissive niche (5) and are critical for the dissemination of the bacteria in the lung, spreading the infection from the alveoli to the interstitium (6). Here, *M. tuberculosis* infects other cell types such as neutrophils, monocyte-derived macrophages, and dendritic cells (DCs). Since DCs present antigens via major histocompatibility complex (MHC) classes I and II to T cells, they function as a connection between the innate and adaptive immune systems (7, 8). Upon antigen presentation to T cells, CD4^+^ T cells produce interferon gamma (IFN-γ), which is involved in the enhancement of macrophage killing and plays an important role in granuloma formation (9). Indeed, in mice (10) and humans (11), the loss of IFN-γ or its receptors, acting as a single nonredundant factor, leads to TB disease. In order to spread to new individuals, *M. tuberculosis* needs to cause pulmonary lesions (12). Inflammation driven by interleukin-1 (IL-1) contributes to host resistance to *M. tuberculosis* (13, 14). Mice that lack IL-1 receptor 1 (IL-1R1), IL-1α, or IL-1β display high susceptibility to *M. tuberculosis* infection (15), with uncontrolled bacterial replication in the lungs, again demonstrating the nonredundant role of one key host pathway.

We hypothesized that bacterial translocation from the phagosome to the cytosol might also be regulated by IL-1. *In vitro*, *M. tuberculosis* can translocate from the phagolysosome to the cytosol (16–23) in an ESX-1-dependent manner (16, 21, 22). This system is responsible for the secretion of a number of proteins, including EsxA (ESAT-6) and EsxB (CFP-10) (reviewed in reference 24). When this secretion system is not present, as is the case for Mycobacterium bovis BCG, cytosolic localization is abrogated in *in vitro* macrophage systems, rendering the bacteria restricted in a membrane-enclosed phagolysosome. Reintroducing the extended *esx-1* locus in BCG allowed translocation to the cytosol and increased virulence, providing clear evidence for an essential role of this type VII secretion system in escape (16, 25–28). In addition, it has been shown that virulent *M. tuberculosis* can form cords in the cytosol and not in the phagosome in human lymphatic endothelial cells *in vitro* (23). This cording is dependent on the ESX-1 secretion system and phthiocerol dimycocerosate (PDIM) glycolipids. The formation of cords in the cytosol rather than in phagosomes suggests a permissive environment for bacterial replication in the cytosol. When *M. tuberculosis* is present in the cytosol, its bacterial DNA is sensed by cyclic GMP-AMP synthase (cGAS) (29–32). This detection is dependent on the presence of a functioning ESX-1 system, suggesting that it is dependent on pathogen-induced cytosolic localization. Cytosolic bacteria colocalize with cytosolic ubiquitin, while this is not the case when the mycobacteria are present in the phagosome (16). Another factor involved in the cytosolic localization is Rv3167c, which regulates the escape of *M. tuberculosis* from the phagosome, since a mutant unable to produce this protein (M. tuberculosisΔRv3167c) displayed increased cytosolic escape (33). Other virulence factors involved in escape from the phagosome are PDIM glycolipids located on the outer membrane of *M. tuberculosis* (19, 25, 34). Mycobacterial strains that lack PDIM are less capable of damaging the phagosomal membrane, resulting in less *M. tuberculosis* in the cytosol of THP-1 macrophages. Recently, it was shown that phagosomal rupture causes the activation of NLRP3-dependent IL-1β release and pyroptosis, a programmed form of cell death (35), facilitating the spread of bacteria to other cells.

While most studies focused on *in vitro* experiments using cultured macrophages, the subcellular localization of *M. tuberculosis* and the factors affecting cytosolic localization and pathogenesis are less intensively studied *in vivo*. When macrophages purified from bronchoalveolar lavage fluids from TB-infected patients were analyzed by electron microscopy (EM), it was found that *M. tuberculosis* is primarily localized in phagosome-like compartments (36, 37). The phagosomal localization does not affect the ability of *M. tuberculosis* to proliferate, and it has long been known that when *M. tuberculosis* is located in phagosomes, it is able to arrest its maturation (38, 39) up to 5 to 7 days (40). More recently, it was shown *in vivo* that *M. tuberculosis* is able to translocate to the cytosol as early as 3 h postinfection using a Förster resonance energy transfer (FRET)-based detection system (41). This study also demonstrated that the pH of the lysosomes plays a role in cytosolic localization: when the lysosome is more acidic, less *M. tuberculosis* is present in the cytosol at 3 days postinfection (dpi) (41).

To examine whether activation via adaptive or innate immunity pathways would affect the ability of mycobacteria to translocate to the cytoplasm *in vivo*, we tested the subcellular localization of different mycobacterial species in zebrafish, armadillo, and mouse models as well as in patient material. In adult zebrafish and zebrafish embryos, we used Mycobacterium marinum, a close homologue of *M. tuberculosis* that is also known to escape the phagolysosome in an ESX-1-dependent manner (42). M. leprae is also known to escape to the cytosol (22), probably using a similar mechanism, and although some of the members of the ESX-1 system (like *esxC*, *esxG*, and *esxS*) are pseudogenes, it has functional ESAT-6 (*esxA*) and CFP-10 (*esxB*), the two most important components needed for cytosolic escape. We used both skin biopsy specimens of leprosy patients and the armadillo model for M. leprae since the armadillo model is known to exhibit the entire clinical spectrum of leprosy (43). For *M. tuberculosis*, both severe combined immunodeficiency (SCID) mice that lack both T and B cells and IL-1R1 knockout mice were compared to determine their ability to limit cytosolic escape *in vivo* in infected cells. Here, we show that while cytosolic localization is limited by both innate and adaptive immunity, IL-1 seems to be a key effector pathway in controlling the cytosolic translocation of *M. tuberculosis*.

## RESULTS

### The pH of the phagosome and lysosome does not affect the cytosolic localization of M. marinum.

**FIG 1:**
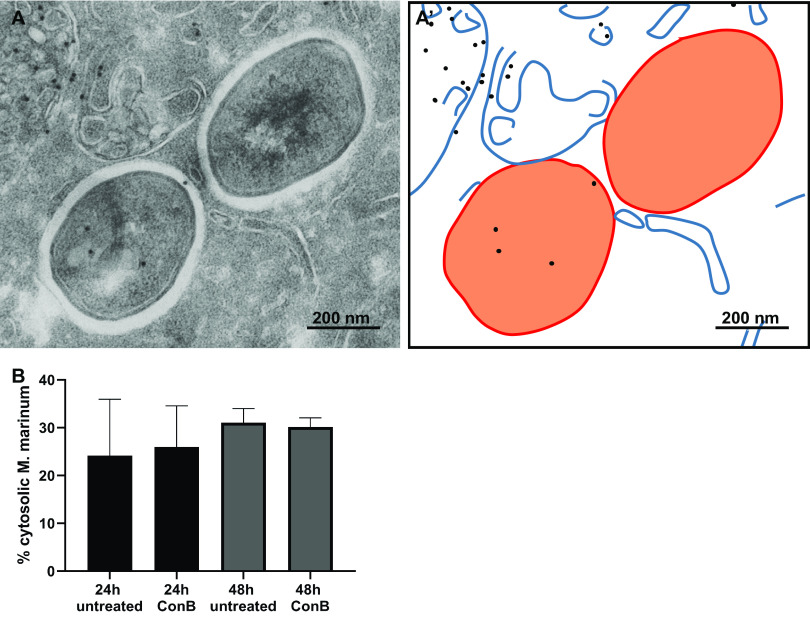
The pH of the phagolysosome does not affect the cytosolic translocation of M. marinum. (A) Electron micrograph of a THP-1 cell infected with M. marinum for 24 h in the presence of ConB showing M. marinum in the cytosol without membrane and CD63 labeling. CD63 immunolabeling indicated by 10-nm gold particles is present on the multivesicular lysosome in the top left corner. (A′) Schematic representation of the micrograph in panel A, with blue lines representing host membranes, black dots representing CD63 labeling indicated by 10-nm gold particles, and orange representing bacteria. (B) Quantification of the percentage of M. marinum bacteria in the cytosol 24 and 48 h after infection using immunogold labeling for CD63 (see also Fig. S2A in the supplemental material). Cells were treated with ConB to raise the lysosomal pH, which did not affect the percentage of cytosolic M. marinum bacteria. Error bars represent the standard deviations from 3 experiments.

*M. tuberculosis* blocks the maturation and acidification of the phagolysosome, promoting its intracellular survival (38, 39, 44–46). Phagosomal acidification is essential for the increased activity of the lysosomal digestive process and, thus, for the degradation of its content (47). *M. tuberculosis* partially avoids acid-mediated killing by blocking fusion between lysosomes and phagosomes (36, 39) and the secretion of an antacid known as 1-tuberculosinyladenosine (TbAd) (48). In addition, it has been shown that the mycobacterial cell wall plays a role in resistance to acidic environments (reviewed in reference 49). We hypothesized that cytosolic escape is a fourth mechanism to avoid lysosome-mediated killing. To determine whether these mechanisms are interdependent, we examine if the phagosomal pH affects translocation from the phagosome to the cytosol. To exclude the effect of TbAd, we utilized M. marinum, which does not express TbAd (50) in THP-1 cells. The acidity of the phagosome and the lysosome was measured by using Lysotracker and by incubation with *N*-{3-[(2,4-dinitrophenyl)amino]propyl}-*N*-(3-aminopropyl)methylamine (DAMP), a weakly basic amine that will be taken up by acidic organelles in live cells (36). After fixation and sample preparation, DAMP was visualized by transmission electron microscopy (TEM) using immunogold labeling with antidinitrophenol (anti-DNP) antibody conjugated to a gold particle. The more acidic the phagosome or lysosome, the more DAMP was present, thus resulting in a higher label density in acidic organelles. As expected, upon M. marinum infection of THP-1 cells, small amounts of DAMP labeling were observed surrounding cytosolic M. marinum (see Fig. S1A and A′ in the supplemental material), while more labeling was detected in M. marinum-containing phagosomes (Fig. S1A and A″). We next blocked acidification with 10 nM concanamycin B (ConB), an inhibitor of vacuolar ATPases that prevents the acidification of endosomes and lysosomes. When cells were treated with ConB and infected with M. marinum, both a lower label density was measured and the Lysotracker imaged by fluorescence microscopy confirmed that the pH is less acidic in the phagosome/lysosome when treated with ConB, as already well described (36, 51) (Fig. S1B). At 1, 24, and 48 h of infection, the cells were lysed, and the number of CFU per well was calculated. At 48 h, the number of bacteria in untreated cells increased 25-fold, compared to that at 0 h, while the number of bacteria in cells treated with ConB increased 9-fold. Thus, in untreated cells, the increase in viable bacteria is higher than the increase in viable bacteria in ConB-treated cells, but M. marinum is still replicating. We next examined whether an increasing pH would affect the efficiency of translocation of M. marinum to the cytosol. The percentage of cytosolic bacteria in THP-1 cells treated with ConB or the no-treatment control was determined both 24 h and 48 h after infection with M. marinum, which is the known time frame for escape (16) (Fig. 1A and B). The numbers of bacteria present in CD63-labeled compartments (phagolysosomal), membrane-enclosed but not CD63-positive compartments (phagosomes), and the cytosol were counted (Fig. S2A). Both 24 h and 48 h after infection, no difference in the percentage of cytosolic bacteria was detected for untreated or ConB-treated cells. This indicates that a higher pH has no effect on M. marinum translocation to the cytosol. In conclusion, a raised lysosomal pH has no effect on the percentage of bacteria translocating to the cytosol in THP-1 cells.

10.1128/mSphere.00153-21.1 FIG S1 The pH of the phagolysosome does not affect the cytosolic translocation of M. marinum. (A) M. marinum-infected THP-1 cells were incubated with DAMP to identify acidic organelles. Thereafter, the cells were fixed and analyzed using TEM. The acidity of the lysosome was determined using immunogold labeling against DAMP using DNP. The higher the label density, the more acidic the phagosome or lysosome. (A′) Cytosolic M. marinum without membranes enclosing the mycobacteria. (A″) Lysosomal M. marinum enclosed by the host membrane and DAMP labeled. (B) Label density as measured by the number of gold particles per square micrometer on M. marinum-containing phagosomes or lysosomes in M. marinum-infected THP-1 cells, M. marinum-infected ConB-treated THP-1 cells, and uninfected untreated THP-1 cells. Download FIG S1, PDF file, 0.3 MB. Copyright © 2021 van der Niet et al. 2021 van der Niet et al. https://creativecommons.org/licenses/by/4.0/ This content is distributed under the terms of the Creative Commons Attribution 4.0 International license.

**FIG 2:**
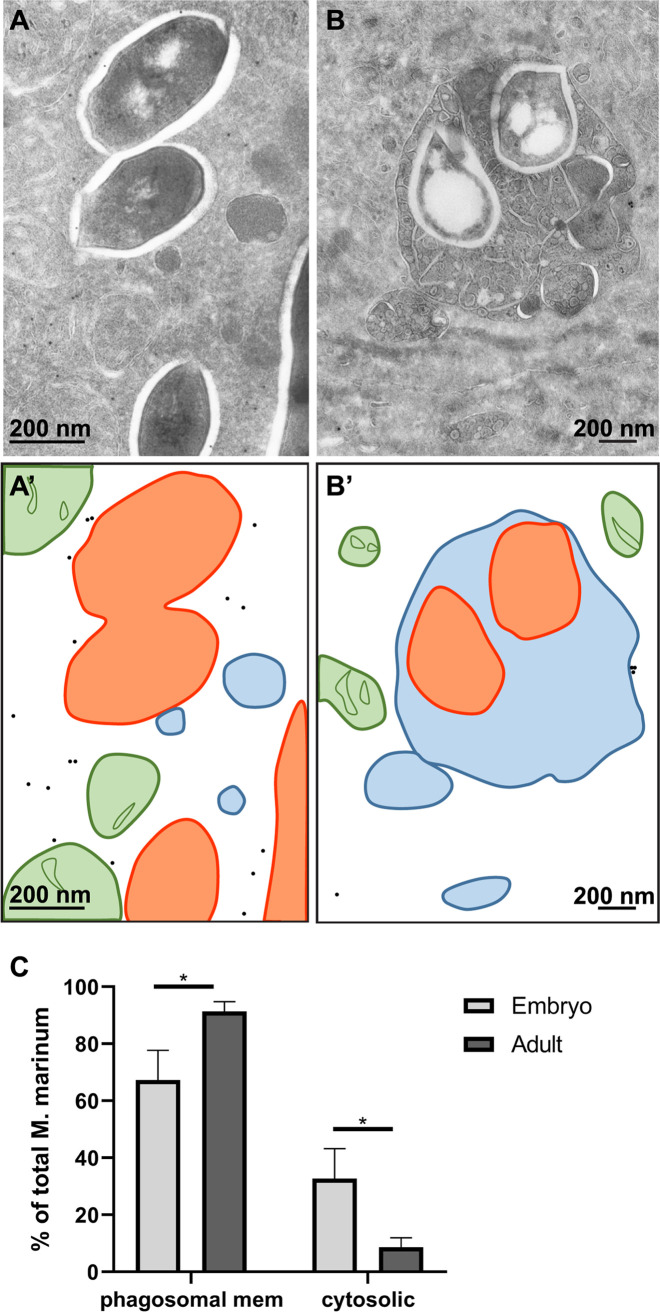
The cytosolic localization of M. marinum in zebrafish is abundant when the adaptive immune system is not yet developed. Embryo and adult zebrafish infected with M. marinum were analyzed using TEM. (A) Cytosolic M. marinum in zebrafish embryo tissue. (B) Phagosomal M. marinum ΔESX-5 bacteria in adult zebrafish tissue. (A′ and B′) Schematic representations of panels A and B, with black dots indicating actin immunogold labeling, orange lines indicating M. marinum, blue lines indicating phagosomal and host membranes, and green lines indicating mitochondria. (C) Quantification of the percentage of cytosolic M. marinum bacteria at embryo day 9 and in the spleen of adult zebrafish at day 11 (see also Fig. S2B in the supplemental material). Error bars indicate standard deviations between 3 different zebrafish embryos and 3 adult fish, and *n* represents the total number of bacteria counted.

10.1128/mSphere.00153-21.2 FIG S2 Quantification of the percentage of mycobacteria in the phagosome, phagolysosome, or cytosol using immunogold labeling and TEM analysis. The bacteria are classified as phagolysosomal when a host membrane is immunogold labeled with at least 2 gold particles conjugated to lysosomal markers such as CD63, LAMP1, or cathepsin D; phagosomal when fewer than 2 gold particles marked the membrane-enclosed compartment; and cytosolic when both membrane and gold are absent. (A) Subcellular localization of M. marinum in THP-1 cells treated with ConB or left untreated. Immunogold labeling with CD63 was performed. Data are averages from 3 experiments with 100 to 200 bacteria classified (Fig. 1B). (B) Subcellular localization of M. marinum in zebrafish embryos on day 9 and in the spleen of adult zebrafish on day 11. Error bars indicate standard deviations between 3 different zebrafish embryos and 3 adult fish. In three embryonic zebrafish, 17, 54, and 74 bacteria were detected, and in three adult zebrafish, 91, 8, and 150 bacteria were detected and categorized. As no good lysosomal markers are present, discrimination between phagolysosomal and phagosomal bacteria cannot be made (Fig. 2). (C) Subcellular localization of M. leprae in skin biopsy specimens of 4 different leprosy patients, using cathepsin D as a lysosomal marker. The error bar indicates the standard deviation from 4 different patients, where 165, 248, 49, and 307 individual bacteria were detected and classified for their subcellular localization (Fig. 4C). (D) Subcellular localization of *M. tuberculosis* in the lungs of WT B6 mice and *Il1r1*^−/−^ mice infected for 4 weeks using LAMP1 as a lysosomal marker (Fig. 6D). Error bars represent standard deviations based on the analysis of 897 (WT) or 618 (*Il1r1*^−/−^) bacteria in multiple granulomas of 2 WT B6 and 2 *Il1r1*^−/−^ mice. Download FIG S2, PDF file, 0.2 MB. Copyright © 2021 van der Niet et al. 2021 van der Niet et al. https://creativecommons.org/licenses/by/4.0/ This content is distributed under the terms of the Creative Commons Attribution 4.0 International license.

### M. marinum translocates to the cytosol in embryonic but not adult zebrafish.

After studying the effect of pH on the ability of M. marinum to translocate to the cytoplasm *in vitro*, we next focused on the subcellular localization *in vivo*. To address whether either adaptive or innate immunity was required to limit bacterial cytosolic escape, we used a zebrafish model combined with M. marinum infection. Among other age-related differences, zebrafish larvae do not have a fully developed adaptive immune system, in contrast to adult zebrafish (52). Zebrafish embryos and adult zebrafish were infected with M. marinum or an M. marinum Tn::ESX-5 mutant, and tissue was fixed for TEM analysis. For analysis of adult zebrafish, a specific M. marinum Tn::ESX-5 mutant was used as this infection was shown to cause hypervirulence (53), and thus, large amounts of bacteria can be detected *in vivo* using TEM. The following conditions were analyzed using TEM: whole zebrafish embryos at day 9 and the spleen of adult zebrafish at day 11 (Fig. 2A and B). In zebrafish embryos, we previously demonstrated that injected M. marinum with fluorescent tags is present in phagocytic cells in the proximity of blood vessels and endothelial cells of these blood vessels (54). Here, we injected the zebrafish with untagged M. marinum and detected bacteria in similar phagocytic and endothelial cells, close to or part of the blood vessels and in phagocytic cells spread throughout the tissue. In these cells, the percentage of cytosolic M. marinum bacteria was determined by counting the number of mycobacteria in a membrane-enclosed compartment and the cytoplasm. Exclusively mycobacteria that were present inside a cell were counted. This is based on the presence of cellular organelles and ribosomes and the electron density of the cytosol or disrupted structures in the extracellular space. Discrimination between phagosomes and phagolysosomes based on immunogold labeling with lysosomal markers available in humans or mice (CD63 and lysosome-associated membrane protein 1 [LAMP1]) was not possible as no lysosomal or cellular markers are available for immunogold labeling in zebrafish. Alternatively, actin antibodies were used as a cellular cytoskeleton marker. In embryos infected for 9 days, 32% of M. marinum bacteria were present in the cytosol (Fig. 2 and Fig. S2B). Noteworthy, ESX-1 mutants remain restricted in the lumen of the blood vessels and are not able to pass the basal lamina of the blood vessel wall (54). As only part of the ESX-1 mutant bacteria are intracellular and the majority are restricted in the lumen of the vessels, the ESX-1 mutants were not included in this study. Moreover, there is ample literature on the ESX-1 localization in membrane-enclosed compartments (16, 22, 25–28, 35). Out of the four adult zebrafish analyzed, one was discarded as no bacteria could be detected. In the other three fish, ample bacteria were detected and categorized as phagosomal or cytosolic based on the presence of membrane. In adult zebrafish, <5% of the bacteria were found in the cytosol (Fig. S2B). Thus, adult but not embryo zebrafish were able to limit bacterial cytosolic escape. This outcome suggests that the presence of an adaptive immune system promotes bacterial containment in the phagolysosome, although other age-related factors could be at play.

### Cytosolic localization of M. leprae is restrained in both armadillo and patient skin.

**FIG 3:**
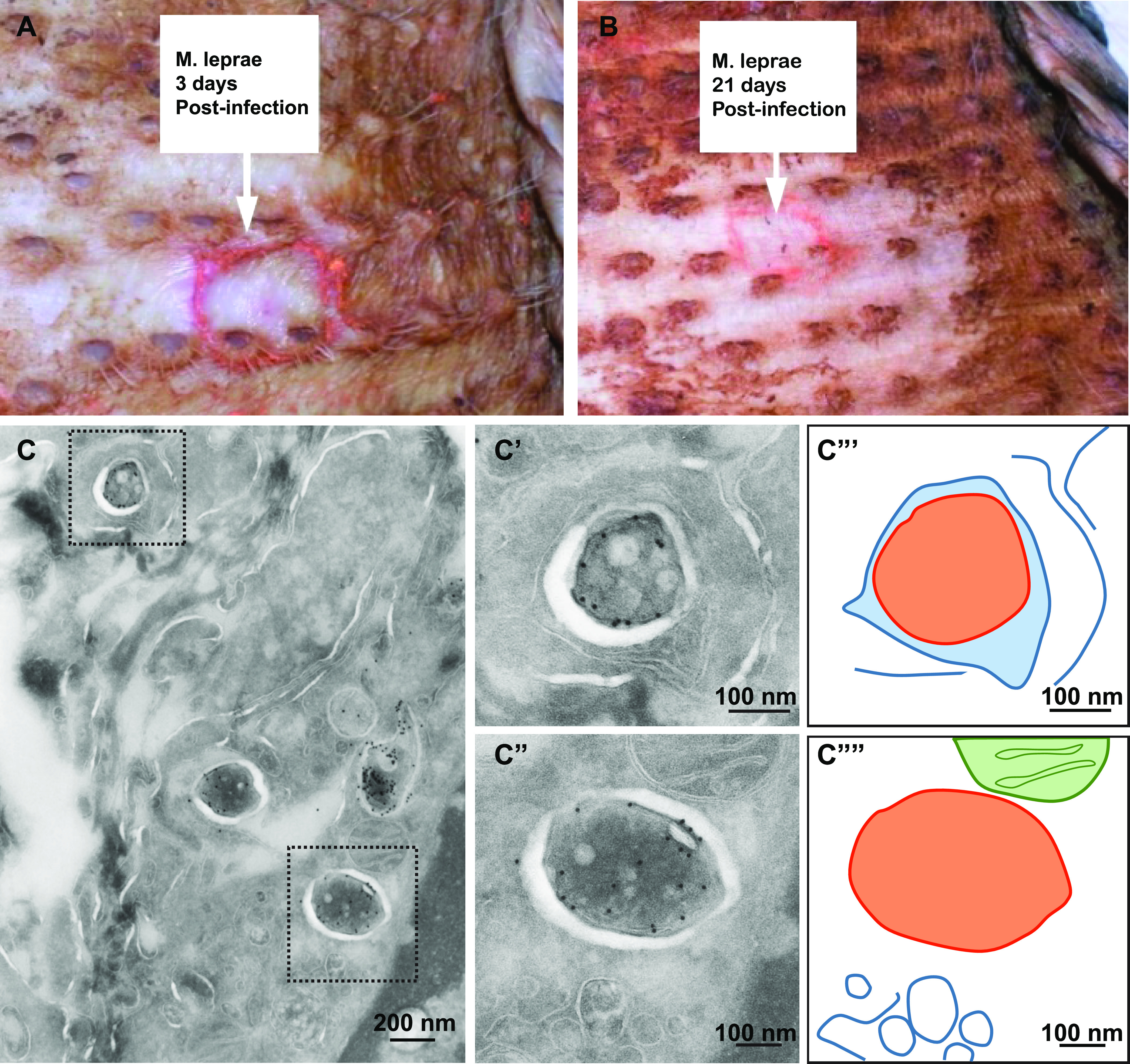
Restrained cytosolic M. leprae localization in armadillo skin biopsy specimens. (A and B) Live M. leprae bacteria were injected into the skin of the armadillo at the red circle indicated by the white arrow text box. The skin was observed 3 days after infection (A) and 21 days after infection (B). At infection sites, loss of pigmentation was detected. (C) TEM image of an infected armadillo skin biopsy specimen 3 days after infection with viable M. leprae. Immunogold labeling using anti-CWP was used to indicate M. leprae. (C′) Enlargement of the upper boxed area in panel C. M. leprae is enclosed by host membranes and is thus phagosomal. (C″) Enlargement of the lower boxed area in panel C. M. leprae was not enclosed by host membranes and was thus cytosolic. (C‴ and C⁗) Schematic representations of panels C′ and C″, with orange indicating M. leprae, the blue lines indicating host membranes, and green indicating mitochondrial membranes. The total numbers of bacteria detected were 47 on day 3 and 45 on day 21.

**FIG 4:**
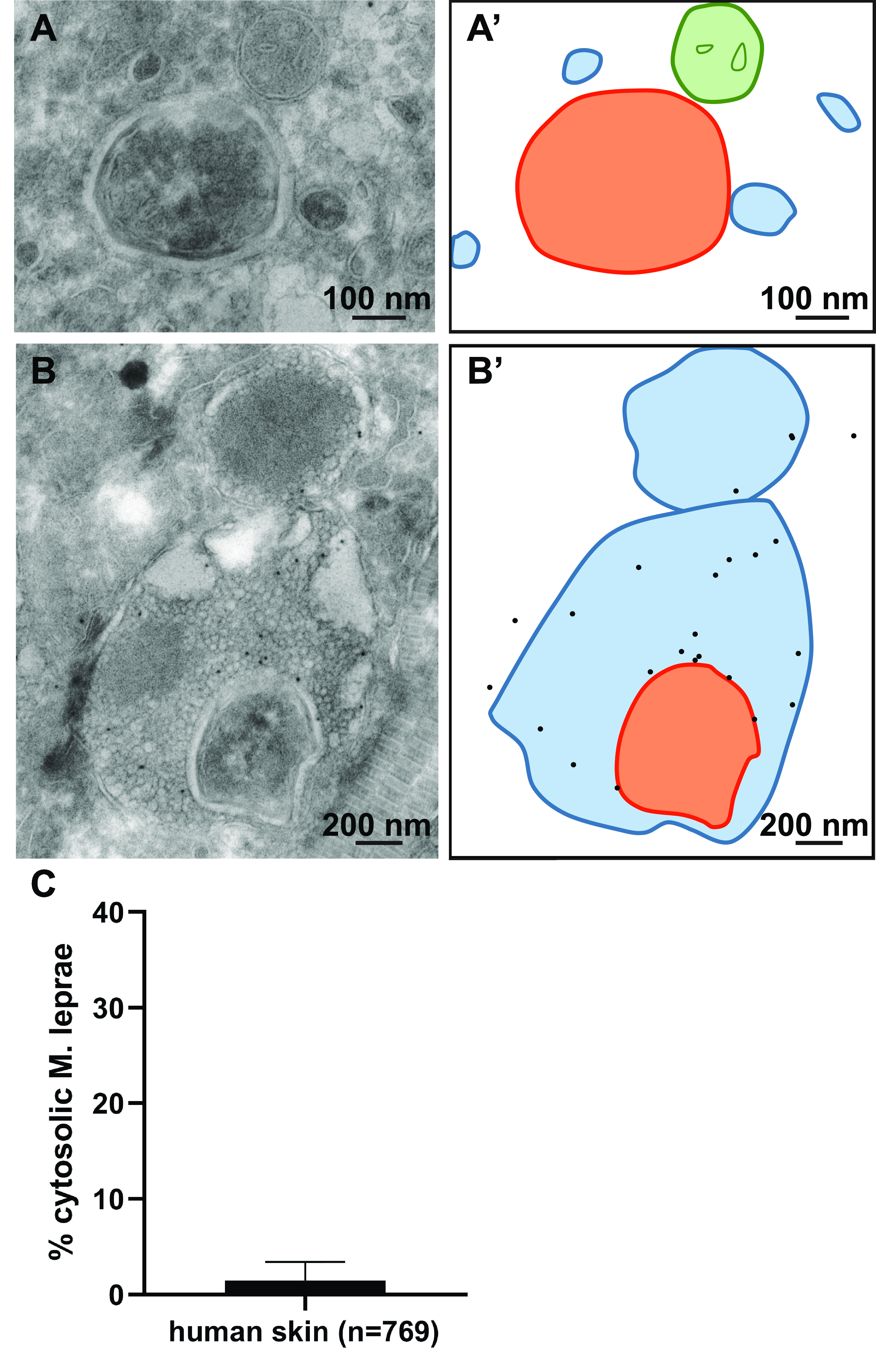
Restrained cytosolic localization of M. leprae in human skin biopsy specimens. Leprosy patient skin biopsy specimens were analyzed using TEM. Immunogold labeling for cathepsin D was used to label lysosomes and phagolysosomes. (A) TEM image of a patient skin biopsy specimen with cytosolic M. leprae. The mycobacteria were not enclosed with host membranes. (A′) Schematic representation of panel A. Orange indicates M. leprae, blue lines indicate host membranes, and green indicates mitochondria. (B) M. leprae present in the phagolysosome. The mycobacterium is enclosed by host membranes and immunogold labeled for the lysosomal marker cathepsin D. (B′) Schematic representation of panel B. Black dots indicate cathepsin D labeling, orange indicates M. leprae, and the blue lines indicate phagolysosomal membranes. (C) Quantification of the average percentage of M. leprae bacteria present in the cytosol. The error bar indicates the standard deviation from 4 different patients, and *n* represents the total number of intracellular bacteria (see also Fig. S2C in the supplemental material).

To examine whether cytosolic localization can be detected only in early, innate stages of infections, we used another model organism, M. leprae, the causative agent of leprosy. Like *M. tuberculosis*, these bacteria have been shown to translocate to the cytosol in an *in vitro* model (22). We studied early-stage (day 3) and late-stage (day 21) infections in armadillos, and in addition, skin biopsy specimens were taken from 4 lepromatous leprosy (LL) patients with a well-established infection. The skin of the abdomen of armadillos was infected with both unviable (irradiated) as well as viable M. leprae. At the site of infection, loss of pigment was visible, which increased during infection progression (Fig. 3A and B). No differences were observed in the loss of pigment between the sites infected with irradiated M. leprae and those infected with viable M. leprae. At the border of pigment loss, armadillo skin biopsy specimens infected with viable M. leprae were fixed for TEM analysis to determine if the localization of the bacteria is cytosolic versus phagosomal. Immunogold labeling against M. leprae-specific anti-cell wall protein (CWP) was used to verify that these are indeed mycobacteria. Similar to the localization of M. marinum in adult zebrafish, the majority of the bacteria were present in membrane-enclosed phagosomes, and cytosolic bacteria were only occasionally detected at both day 3 (Fig. 3C) and day 21 after infection. In addition, skin biopsy specimens of 4 different lepromatous leprosy patients taken from the border of the infection as defined by the depigmentation line were analyzed using TEM and immunogold labeling for M. leprae-specific anti-cell wall protein and lysosomal markers such as cathepsin D (Fig. 4 and Fig. S3), LAMP1, and CD63. Worth mentioning is that based on the ultrastructure of the cells, and more specifically the ultrastructure of the cell nucleus or the presence of multiple lysosomes, most bacteria reside in macrophage-like cells. From the 4 patients, individual bacteria were detected and classified for their subcellular localization based on the presence of a surrounding membrane and 2 or more gold particles detecting cathepsin D (Fig. S2C). Interestingly, the ultrastructure of the bacteria in the phagosome is damaged, with an amorphous shape and an irregular capsular layer (Fig. S3), whereas the cytosolic bacterium has a regular intact appearance. Similar to the armadillo and adult zebrafish, the percentage of cytosolic mycobacteria was low (1.1%), while over 700 bacilli were assessed. In conclusion, in both armadillo and human skin, a low percentage of cytosolic M. leprae bacteria is present at all measured stages of infection.

10.1128/mSphere.00153-21.3 FIG S3 (Top) Electron micrograph of the skin biopsy specimen of a leprosy patient showing M. leprae in the cytosol without a membrane and clusters of degraded M. leprae bacteria in phagosomes with CD63 labeling. CD63 immunolabeling indicated by 10-nm gold particles is present on the phagolysosomes. (Bottom) Schematic representation of the micrograph in the top panel, with blue lines indicating host membranes, orange bacteria with dotted lines indicating bacteria showing signs of degradation, and continuous lines indicating an intact bacterium. Bar, 500 nm. Download FIG S3, PDF file, 0.3 MB. Copyright © 2021 van der Niet et al. 2021 van der Niet et al. https://creativecommons.org/licenses/by/4.0/ This content is distributed under the terms of the Creative Commons Attribution 4.0 International license.

### Immunocompetent BALB/c but not SCID mice can contain M. tuberculosis inside phagosomes of infected pulmonary cells.

**FIG 5:**
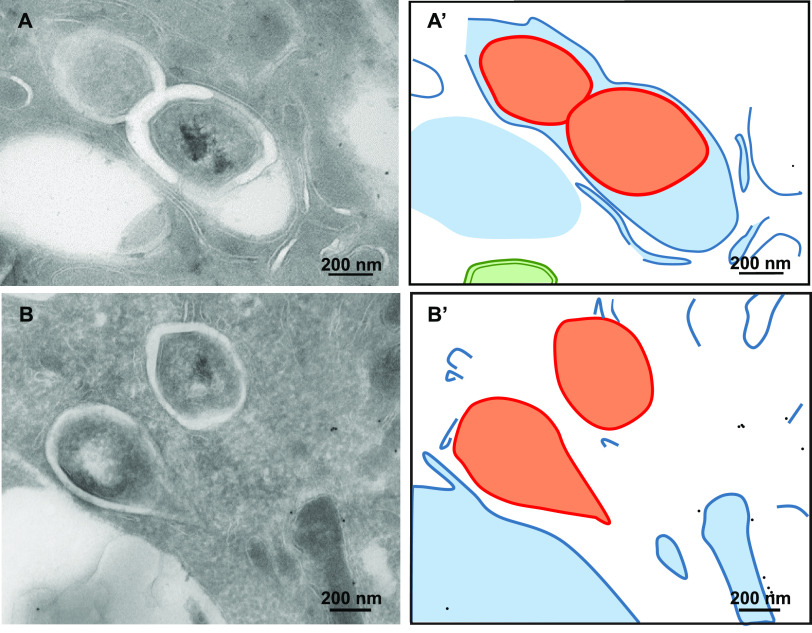
Early cytosolic localization of *M. tuberculosis* in SCID mouse lungs. (A and B) Sections of SCID mouse lung tissue infected with *M. tuberculosis* H37Rv for 21 days were labeled for LAMP1 using immunogold labeling to indicate lysosomes. (A′ and B′) Schematic representations, with orange indicating *M. tuberculosis*, blue lines indicating host membranes, black dots representing gold particles indicating LAMP1-decorated lysosomes, and green indicating mitochondria. Panel A shows a cross section of *M. tuberculosis* surrounded by cellular membranes, which is thus phagosomal. Panel B shows *M. tuberculosis* present in the cytosol, without cellular membranes surrounding the bacteria. For information on the number of imaged bacteria and mice, see Table S1 in the supplemental material.

After showing that neither M. leprae nor M. marinum translocates in high numbers to the cytosol even at later stages of infection, we next wanted to study the subcellular localization of M. tuberculosis longitudinally in mice. To do so, BALB/c mice were infected with the virulent *M. tuberculosis* strain H37Rv, and lung tissue was fixed for EM analysis at days 2, 7, 21, 45, and 120 after infection. Based on the ultrastructure of the nucleus and/or the high number of lysosomes, the intracellular bacteria were detected in macrophage-like cells. These samples were immunogold labeled for lysosomes using LAMP1 or cathepsin D and imaged using TEM (Fig. 5). As for M. marinum in THP-1 cells and M. leprae in skin, the numbers of bacteria present in LAMP1-labeled phagolysosomes, membrane-enclosed but not LAMP1-labeled phagosomes, and the cytosol were counted (Table S1). We found that cytosolic localization was highest at day 7 of infection, with still <5% percent of *M. tuberculosis* bacteria being detectable in the cytosol and most bacteria residing in a membrane-enclosed compartment. To determine if patient-derived strains behave similarly to H37Rv, BALB/c mice were infected with two additional *M. tuberculosis* strains from lineage 2, often referred to as Beijing family *M. tuberculosis* (1998-1500 ancient Beijing and a multidrug-resistant strain [2002-0230 Beijing]). EM analysis was done at 21, 45, and 120 dpi and also demonstrated small amounts of cytosolic bacteria (Table S1). Thus, as shown previously in immunocompetent mice, different strains of *M. tuberculosis* reside primarily inside membrane-enclosed compartments and not inside the cytosol.

10.1128/mSphere.00153-21.4 TABLE S1 Overview of the subcellular localization of various *M. tuberculosis* strains at different days of infection in BALB/c or SCID mice. The numbers of bacteria used and the numbers of mice in which bacteria were detected are given in the last 2 columns. At least 2 mice were always included in the infection experiments and fixed for EM analysis. The numbers of mice in which bacteria were detected and EM classification was performed are presented. Bacteria are classified as phagolysosomal, phagosomal, or cytosolic based on the number of gold particles attached to lysosomal markers and the presence of a membrane. Download Table S1, PDF file, 0.08 MB. Copyright © 2021 van der Niet et al. 2021 van der Niet et al. https://creativecommons.org/licenses/by/4.0/ This content is distributed under the terms of the Creative Commons Attribution 4.0 International license.

To directly address the potential contribution of the adaptive immune system, we next quantified *M. tuberculosis* cytosolic translocation in SCID mice. SCID mice lack functional T and B cells, key mediators of adaptive immunity in vertebrates, and succumb rapidly to *M. tuberculosis* infections. SCID mice were infected with *M. tuberculosis* strain H37Rv via aerosol and sacrificed 21 days after infection. Lungs were then fixed and processed for EM analysis, and sections were labeled for LAMP1 using immunogold labeling to indicate lysosomes and phagolysosomes (Table S1). We detected a 10-fold increase in *M. tuberculosis* cytosolic translocation in a SCID mouse compared to BALB/c mice, arguing that T and B cells are required for optimal clearance of cells with cytosolic bacilli, although we cannot exclude that in immunocompetent mice, adaptive responses prevent escape.

### *M. tuberculosis* is preferentially located in the cytosol of infected pulmonary cells in IL-1R1-deficient mice.

**FIG 6:**
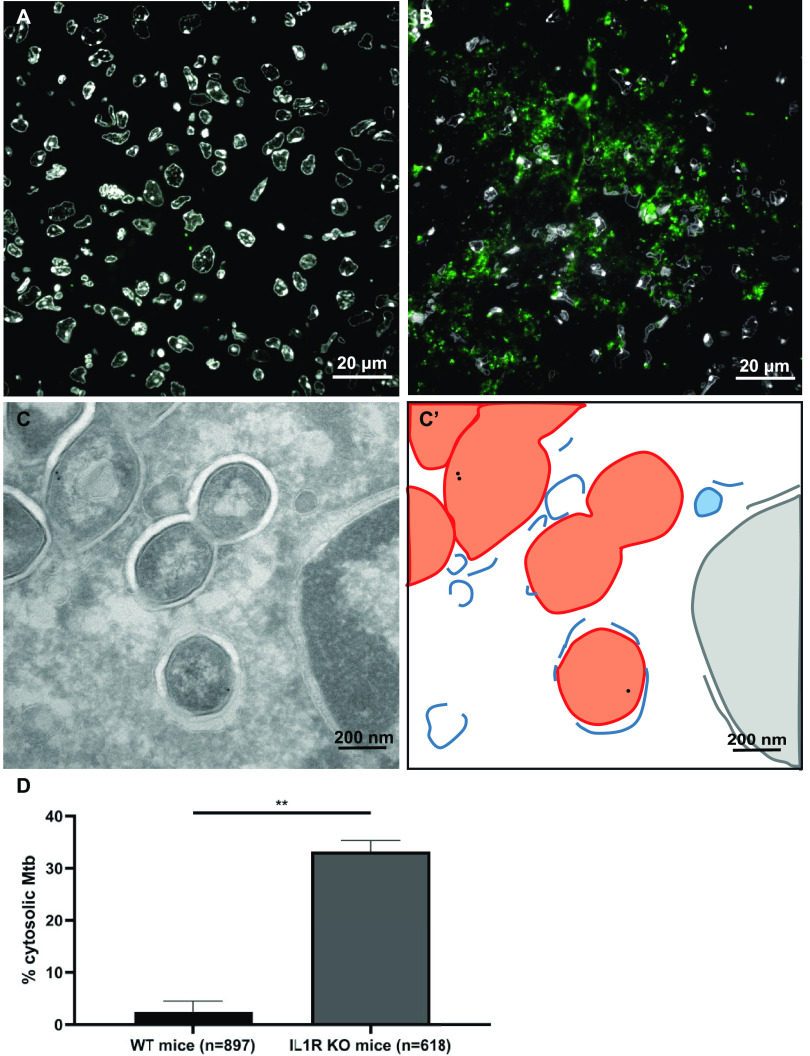
*M. tuberculosis* preferentially localizes to the cytosol in *Il1r1^−/−^* mice. (A and B) Fluorescence microscopy of 200-nm sections stained with DAPI (4′,6-diamidino-2-phenylindole) for nuclei (white) and anti-cell wall protein to detect *M. tuberculosis* (green) in granulomas in lung tissues of WT B6 mice (A) and *Il1r1^−/−^* mice (B) infected with *M. tuberculosis* for 28 days. (C) Immunogold labeling using LAMP1 to indicate lysosomal membranes in *Il1r1^−/−^* mice imaged using TEM. (C′) Schematic representation of panel C. *M. tuberculosis* is depicted in orange, host membranes are in blue, and the host nucleus is in pink. (D) Quantification of the localization of *M. tuberculosis* in B6 and *Il1r1^−/−^* lungs, here presenting cytosolic localization (see also Fig. S2D in the supplemental material). The analysis included 897 (WT) or 618 (*Il1r1^−/−^*) bacteria, and error bars indicate standard deviations based on in multiple granulomas of 2 WT B6 and 2 *Il1r1^−/−^* mice. KO, knockout.

IL-1 is a potent innate inflammatory cytokine critically required for resistance against bacterial infections. Mice deficient in the IL-1 pathway, such as IL-1α and IL-1β, are highly susceptible to *M. tuberculosis* infection, with increased mortality and bacterial growth in the lung and spleen and the development of necrotic granulomatous lesions that more closely resemble human necrotic lesions (14, 15, 55–57). To investigate the contribution of this innate immune pathway to the prevention of mycobacterial cytosolic escape, we infected *Il1r1^−/−^* and B6 wild-type (WT) mice with *M. tuberculosis* and 4 weeks after infection fixed lung tissue and processed it for TEM analysis. As *M. tuberculosis* is normally difficult to find, fluorescence microscopy was first performed, and tissues were sectioned at 200 nm and stained for the detection of nuclei and mycobacteria (Fig. 6A and B) to be able to select for the infected area (54). Whereas in B6 mice, only small spots of *M. tuberculosis* are detected (as for BALB/c mice), infected *Il1r1^−/−^* tissue is heavily labeled and thus heavily infected. Ultrathin sections were labeled for LAMP1 and CD63 using immunogold labeling to indicate lysosomes and phagolysosomes for TEM analysis. To determine if the IL-1 pathway has an effect on the cording of *M. tuberculosis*, this was monitored by determining the average number of bacteria per cluster, but no difference was detected between the cluster sizes in infected *Il1r1^−/−^* and B6 tissues. Similar to the localization of M. leprae in skin biopsy specimens and *M. tuberculosis* in BALB/c mouse tissue, *M. tuberculosis* is mainly detected in macrophage-like cells. The identification of the cells as macrophages is based on morphological characteristics such as the typical nucleus and presence on lysosomes as well as ionized calcium binding adaptor molecule 1 (iba1) labeling. As before, the difference between phagolysosomal, phagosomal, and cytosolic *M. tuberculosis* was determined based on the presence of immunogold labeling and a membrane surrounding the bacteria (Fig. 6C and D and Fig. S2D). *M. tuberculosis* was found in the phagolysosome, the phagosome, and the cytosol. Strikingly, we detected a 14-fold increase in the number of *M. tuberculosis* bacteria that translocated to the cytosol in *Il1r1^−/−^* lungs compared to WT B6 lungs infected with *M. tuberculosis*. While only 2.5% of *M. tuberculosis* bacilli were located in the cytosol in the lungs of infected WT B6 mice, over 30% of the *M. tuberculosis* bacilli in the lung were able to escape to the cytosol in the absence of IL-1 signaling. Thus, IL-1-dependent signals are required to control the cytosolic localization of *M. tuberculosis.*

## DISCUSSION

The cytosolic localization of mycobacteria has been debated ever since the first description in the late 1980s (18, 20, 58). Our immunogold TEM analysis of *M. tuberculosis*, BCG, and mutant strains in 2007 (22) restarted the discussion, and several studies have now confirmed the presence of extraphagosomal *M. tuberculosis* bacilli (16, 21, 41). In addition, the release of DNA into the cytosol was described and is, like escape from the phagosomal compartment, dependent on the ESX-1 secretion system (29, 31, 32). In this study, we showed that *in vivo*, and with an intact immune response, *M. tuberculosis* is present mainly in membrane-bound compartments and not in the cytosol of infected cells. This is also true for the pathogenic mycobacteria M. leprae and M. marinum.

Here, we showed that raising the lysosomal pH did not affect the cytosolic localization of M. marinum. In THP-1 cells, M. marinum can escape irrespective of the raised pH of the lysosome. As already well known, maturation of the *M. tuberculosis* phagosome is altered (38, 44, 45), and our data suggest that the pH of phagosomes has no effect on translocation. Simeone and colleagues (41) showed *in vivo* that when phagosomal acidification was blocked using bafilomycin, an induction of mycobacterial access to the cytosol is detected. Therefore, we hypothesized that raising the phagosomal and lysosomal pH by ConB would result in increased cytosolic localization of M. marinum. However, we found, at a cellular level, no difference in the percentage of cytosolic M. marinum bacteria. Our cell culture-derived data demonstrate merely that mycobacteria can translocate irrespective of the pH, and thus, manipulation of the pH by ConB did not change the subcellular localization in cells under *in vitro* conditions. Importantly, *in vivo*, as studied here and by Simeone et al. (41), subcellular localization of mycobacteria is a highly complex and dynamic process.

After inhalation in the lung, *M. tuberculosis* is initially taken up by alveolar macrophages and spreads among innate immune cells while delaying the initiating adaptive immunity (59–61). Importantly, both innate and adaptive immunity are critically important for optimal host resistance against *M. tuberculosis*. Here, we show that while the lysosomal pH itself has a minimal role, effective adaptive and innate host immunity play a critical role in regulating the number of cells with cytosolic mycobacteria. The delicate balance between bacterial replication and containment by the host immune response is lost when immunity is compromised by either the lack of T and B cells in SCID mice or, even more dramatically, the absence of innate IL-1R1 signaling. Of note, the profound increase in IL-1R1-deficient mice exceeds the moderate but significant increase in SCID mice, even though the former likely has a broader set of immunological defects. This may argue that despite increased susceptibility to *M. tuberculosis*, certain specific immune pathways like IL-1 may be more directly and preferentially involved in regulating the clearance of cells containing cytosolic bacteria. It cannot be excluded that in immunocompetent mice, adaptive immune responses prevent bacterial cytosolic escape, although no mechanism is known. It is known that the activation of cytosolic immunosurveillance pathways, including those linked to IL-1, promotes rapid clearance by the immune cells attracted to infected cells.

Cytosolic immunosurveillance pathways are triggered by pathogens directly or by pathogenic products entering the cytosol and are often linked to antiviral immunity and type I IFN induction as well as inflammasome activation. Inflammasomes are cytosolic signaling complexes that ultimately lead to cytolytic cell death and IL-1 family cytokine processing. It is now established that bacterial DNA can translocate to the cytosol, in an ESX-1-dependent manner, where it is detected by cytosolic DNA sensors such as cGAS and AIM-2 (27, 29, 31, 32). cGAS in turn synthesizes a second messenger, cyclic guanosine monophosphate-adenosine monophosphate (cGAMP), which activates stimulator of IFN genes (STING) and type I IFN signaling and the expression of IFN-α and -β (reviewed in reference 62). When cytosolic DNA is detected by AIM-2, the NRLP3 inflammasome is activated and leads to the proteolytic cleavage of IL-1β (27, 29, 31, 32). Thus, the translocation of *M. tuberculosis* to the cytosol triggers a cascade of responses resulting at a cellular level in cell death (22, 63, 64). The connection between translocation, cellular damage, and cell death has recently been studied in more detail; plasma membrane lysis caused by *M. tuberculosis* triggers inflammasome activation, IL-1β release, and pyroptosis, a form of programmed necrosis (35). Beckwith et al. (35) demonstrated that ESX-1-mediated phagosomal damage is a requirement for NLRP3 activation, and although their live-cell imaging demonstrated that inflammasome activation by *M. tuberculosis* is independent of lysosomal damage, others have demonstrated that active cathepsin release from ruptured phagolysosomes is a trigger of NLRP3 inflammasome activation (64). Taken together, after rupture of the phagolysosomes by *M. tuberculosis*, cell death is induced, and with an intact immune response, via IL-1 signaling, these cells are cleared. This explains the fact that in skin biopsy specimens of leprosy patients and armadillos, lungs of BALB/c mice, and adult zebrafish, the majority of the mycobacteria is detected in phagolysosomes. We have now demonstrated that in the skin of leprosy patients, where the infection is well established and possibly ongoing for years, phagolysosomal bacteria are degraded, while cytosolic bacteria appear healthy. These effects were not previously detected in *in vitro* systems (16, 19, 22, 23, 35) as these systems do not allow long incubation periods.

We have previously shown that IL-1 and type I IFNs exhibit potent cross-regulation important for host resistance against *M. tuberculosis* with excessive type I IFN induction in the absence of IL-1 signaling (14, 15, 65). The elevated type I IFN expression in the absence of IL-1 contributed to the increased susceptibility of *Il1r1^−/−^* mice, as mice doubly deficient in IL-1R1 and IFNAR1 displayed increased resistance (14). Our new findings here of increased cytosolic *M. tuberculosis* in *Il1r1^−/−^* mice provide a possible molecular explanation for the increased type I IFN production previously reported. In this context, our recent study demonstrated that infected cells themselves do not need to express IL-1R1 *in vivo* to mediate host resistance and that IL-1R1 expression coordinates immune responses in multiple cell types (13). Along these lines, it has been proposed that IL-1R1 on nonimmune cells was required for the ability of infected alveolar macrophages to leave the airway to establish infection in the interstitial lung space (6). Thus, cytosolic containment of bacilli in infected cells may not require direct cell-autonomous antimicrobial signaling pathways but may be the result of dynamic cellular interactions between infected cells and cells of both nonhematopoietic and bone marrow origins.

Overall, this study establishes that high-level cytosolic escape of mycobacteria can indeed occur *in vivo* but mainly when host resistance is compromised. When the complete adaptive immune system, including B and T cells, is abrogated, like in zebrafish embryos and SCID mice, a substantial percentage of mycobacteria is detected in the cytosol compared to immunocompetent hosts. Strikingly, the highest proportion of cytosolic *M. tuberculosis* was observed in mice lacking IL-1 signaling. This argues that the IL-1 pathway is crucial for the control of the number of cytosolic mycobacteria and likely IL-1-mediated resistance to *M. tuberculosis*.

## MATERIALS AND METHODS

### Bacteria.

The M. marinum E11 strain was grown on Middlebrook 7H10 plates supplemented with oleic acid-albumin-dextrose-catalase (OADC). A single colony was inoculated into 7H9 liquid medium (BD) supplemented with 10% albumin-dextrose-catalase (ADC) and 0.05% Tween 80, incubated with shaking at 30°C, and grown to an optical density at 600 nm (OD_600_) of 0.6 to 1. Before infection, the bacteria were centrifuged at 750 rpm to remove clumps, leaving a bacterial suspension.

### Inhibiting acidification.

Inhibition of acidification of phagosomes/lysosomes in THP-1 cells treated with concanamycin B (ConB) (catalogue no. 380098C100; Alexis Biochemicals) was assessed by confocal microscopy. THP-1 macrophages were grown in RPMI 1640 medium supplemented with 10% fetal calf serum (FCS), and ConB was added to the cells at different concentrations 1 h and 24 h before fixation. A concentration of 10 nM ConB strongly inhibited acidification and did not affect THP-1 cell viability after a 48-h incubation period. THP-1 cells were washed 3 times with RPMI 1640–10% FCS medium and kept as a control or pretreated with 10 nM ConB for 1 h. Cells were infected with M. marinum (multiplicity of infection [MOI] of 10:1) in the presence or absence of the acidification inhibitor. After an incubation time of 1 h at 32°C, cells were washed 3 times with culture medium without antibiotics and with or without acidification inhibitors to remove extracellular bacteria. After washing, the cells were further incubated in culture medium with or without ConB for 24 or 48 h at 32°C prior to fixation. Fixed samples were prepared for cryo-immunogold microscopy, sectioned for EM, and immunogold labeled with CD63. To assess the effect on the number of cytosolic M. marinum bacteria, from 3 independent experiments, 100 to 200 randomly chosen bacteria from one grid were counted, and for each bacterium, it was determined if it was cytosolic or resided in a phagolysosome.

### DAMP assay for measuring lysosomal acidification.

Luminal acidification in lysosomes was measured via the probe DAMP [3-(2,4-dinitroanilino)-3′-amino-*N*-methyldipropylamine] incubated at 30 μM for 30 min to allow accumulation in acidic compartments. DAMP was quantified by immunogold staining using anti-DNP.

### Mouse infections.

*IL1r1^–/–^* mice were purchased from Jackson Laboratories (catalogue no. JAX 3018) and backcrossed to C57BL/6 control mice from Taconic Farms (Hudson, NY) for 11 generations. Male and female mice, 8 to 12 weeks of age, were infected via the aerosol route with *M. tuberculosis* H37Rv (100 to 200 CFU/mouse) as previously described (13) and sacrificed 27 days later. In short, mice were infected using a whole-body inhalation system (Glas-Col, Terre Haute, IN) exposing the mice to aerosolized *M. tuberculosis*. Lungs were perfusion fixed in 4% paraformaldehyde and 0.4% glutaraldehyde overnight. After fixation, tissues were transferred to storage buffer containing 0.5% paraformaldehyde. All animals were maintained in Association for Assessment and Accreditation of Laboratory Animal Care (AALAC)-accredited biosafety level 2 (BSL2) or BSL3 facilities at the National Institutes of Health (NIH), and experiments were performed in compliance with an animal study proposal approved by the National Institute of Allergy and Infectious Diseases Animal Care and Use Committee.

### SCID mice.

Severe combined immunodeficiency (SCID) mice were purchased from Charles River and were aerosol infected with M. tuberculosis H37Rv for 21 days, when mice were sacrificed and lungs were fixed by perfusion fixation as described below.

### BALB/c and B6 mice.

Pathogen‐free male BALB/c mice, 6 to 8 weeks old, were anesthetized with sevoflurane vapors (Abbott Laboratories, Abbott Park, IL, USA), and 100 μl of phosphate-buffered saline (PBS) with 2.5 × 10^5^ viable H37Rv bacilli or either Beijing clinical isolate was inoculated intratracheally using a stainless steel cannula. Groups of 15 animals were then maintained in cages fitted with microisolators in a BSL3 facility. Following infection, three mice were euthanized by exsanguination under anesthesia with pentobarbital at days 2, 7, 21, 45, and 120 of infection. Lung tissues were fixed by perfusion fixation as described below.

### Zebrafish.

Zebrafish embryos and adult zebrafish (Danio rerio) were microinjected with M. marinum strain E11 as described previously (53) at 30°C. In short, zebrafish embryos were infected at 28 h postinfection with 100 CFU of wild-type M. marinum through microinjection in the caudal vein. Adult zebrafish were anesthetized in 0.02% MS-222 (Sigma) and injected intraperitoneally with 2 × 10^4^ M. marinum Tn::ESX-5 bacteria. The embryos were incubated for 6 or 9 days and fixed as described below. For classification of the subcellular localization, 3 embryos at 9 days of incubation were used. Three adult fish were infected with E11 or the E11 ESX-5 mutant (53) and sacrificed at day 11, when the spleen was dissected and fixed as described below.

### Armadillo.

Male armadillos were intradermally inoculated in the abdomen with 1 × 10^7^ bacteria of either live or irradiated M. leprae (0.1 ml). At 3, 11, and 21 days postinoculation, 6-mm punch biopsy specimens were taken from the border of the depigmented area at the inoculation sites and fixed for 2 h as described below. For classification of the subcellular localization, 1 to 2 punches were used from day 3 and day 21. As no lysosomal markers suited for immuno-TEM analysis were known, anti-cell wall protein (CWP) was used to immunolabel the leprosy bacteria.

### Human samples.

Leprosy skin biopsy specimens were taken at the border of the depigmented lesions with written consent from 4 different lepromatous leprosy patients. Materials were directly incubated in EM-grade fixatives and transported to the EM laboratory in fixatives.

### Fixation of tissues.

All tissues were fixed in a combination of 4% paraformaldehyde and 0.4% glutaraldehyde in 0.2 M PHEM [240 mM piperazine-*N*,*N*′-bis(2-ethanesulfonic acid) (PIPES), 100 mM HEPES, 8 mM MgCl_2_, and 40 mM EGTA] buffer. Fixation for at least 2 h in a fixative containing glutaraldehyde is essential to kill mycobacteria. After fixation, tissues were transferred to storage buffer containing 0.5% paraformaldehyde in 0.2 M PHEM buffer.

### Embedding and sectioning.

After fixation, tissues were washed in PBS, to remove fixatives. The required structures were dissected using a razor blade and cut into 1- to 3-mm^2^ blocks. Lung tissue was embedded in increasing percentages of gelatin (2%, 5%, and 12% in 0.1 M phosphate buffer) and incubated at 37°C. After removal from liquid gelatin, blocks were incubated overnight in 2.3 M sucrose at 4°C. Next, blocks were snap-frozen and stored in liquid nitrogen until sectioned. After trimming at −100°C, semithin sectioning was performed for analysis by fluorescence microscopy, or ultrathin sectioning at −120°C was performed using a diamond Diatome cryo-immuno knife on a Leica Ultracut UC6 ultramicrotome. Sections were picked up with a loop filled with a 1:1 mixture of 2.3 M sucrose and 1% tylose (methylcellulose [G1095]) in MilliQ water and placed on a copper, Formvar-coated grid or glass slides. Grids with sections were stored at 4°C until immunolabeled. Fluorescence microscopy was used to search the region of infection as described previously (54, 66). In short, semithin sections (200 to 300 nm) of the whole sample were labeled with Hoechst 33342 (Thermo Fisher) to indicate the nuclei of the tissue, and anti-cell wall protein labeling was used to indicate mycobacteria.

### Immunogold labeling.

Grids with cryosections were incubated on 2% gelatin in 0.1 M phosphate buffer plates at 37°C for 30 min. Thereafter, grids were washed with PBS–0.02 M glycine, blocked with 1% bovine serum albumin (BSA), and incubated for 45 min with primary antibody. Various antibodies were used on different tissues: cathepsin B (clone 1C11; Zymed), lysosome-associated membrane protein 1 (LAMP1) and LAMP2 (clones H4A3 and H4B3; Pharmingen), and cluster of differentiation CD63 (clone 435; Sanquin) for human skin and sputum; antiactin (clone AC-15; Sigma) for zebrafish; various antibodies tested but without specific labeling, including anti-LAMP1 (Pharmingen [clones H4A3 and H4B3] and Abcam [catalogue no. ab67283]) and anti-CD63 (clone 435; Sanquin); and anti-cell wall protein (C188, a kind gift from John Spencer and Patrick Brennan, Colorado State University) for M. leprae. *N*-{3-[(2,4-Dinitrophenyl)amino]propyl}-*N*-(3-aminopropyl)methylamine dihydrochloride) (DAMP) detection was performed using antidinitrophenol (anti-DNP) (polyclonal anti-DNP; Oxford Biomedical Research). As a macrophage marker, iba1 (catalogue no. Ab107159; Abcam) was used. All antibodies were diluted in 1% BSA in PBS. After washing in PBS–0.02 M glycine and blocking in 0.1% BSA in PBS–0.02 M glycine, grids were incubated on a bridging antibody when the primary antibody was monoclonal or of goat origin. After washing and blocking, grids were incubated on protein A gold diluted in 1% BSA in PBS. To remove unbound gold, grids were washed with PBS, fixed using 1% glutaraldehyde in PBS, washed with MilliQ water, and contrasted with uranyl acetate and methylcellulose at pH 4.

### Immunofluorescence labeling.

Semithin sections (200 to 300 nm) placed on glass were washed with PBS–0.02 M glycine and incubated with the primary antibody anti-cell wall protein for 45 min. Next, the sections were washed with PBS and incubated with the secondary antibody goat anti-rabbit Alexa Fluor 488 (catalogue no. A32731; Molecular Probes) for 20 min and Hoechst 33342 for 5 min (catalogue no. H3570; Thermo Fisher). After washing with PBS, samples were mounted with Vectashield. The sections were imaged using a Leica DM6 wide-field microscope, and images were analyzed using FIJI.

### Statistical analysis and subcellular classification.

Statistical analysis was performed using GraphPad Prism 8.0 software. In the figure legends, the averages are given with the standard deviations, and *n* indicates the number of bacteria localized in a specific subcellular compartment. Significance was determined by using an unpaired *t* test and is defined in the graphs (*, *P* < 0.05; **, *P* < 0.01; ***, *P* < 0.001). The numbers of biological samples and bacteria counted in mice are listed in Table S1 in the supplemental material.

Classification of the subcellular localization of bacteria was performed blindfolded and by 2 individual counters to establish intercounter reproducibility. Bacteria are classified as cytosolic when 1/3 or less of the bacteria or bacterial cluster is surrounded by a visible membrane and 2 or fewer gold particles detecting lysosomal markers are present. Bacteria are classified as phagosomal when 1/3 or more of the bacteria or bacterial cluster is surrounded by a visible membrane and 2 or fewer gold particles detecting lysosomal markers are present and phagolysosomal when 1/3 or more of the bacteria or bacterial cluster is surrounded by a visible membrane and 3 or more gold particles detecting lysosomal markers are present.

### Ethics statement.

SCID mouse infections were performed in agreement with European and French guidelines (directive 86/609/CEE and decree 87-848 of 19 October 1987). The experiments received approval by the Institut Pasteur Safety Committee (protocol 11.245) and ethical approval by local ethical committee Comité National de Réflexion Ethique sur l’Expérimentation Animale no. 59 (CNREEA).

Adult zebrafish of the local Free University of Amsterdam (VU) line were handled in compliance with local animal welfare regulations and approved by the local animal welfare commission (IvD) of the VU/Amsterdam University Medical Centre. For zebrafish embryo experiments that are performed within the grace period (i.e., the first 6 days), no special permission is allowed since these experiments fall under animal experimentation law according to EU animal protection directive 2010/63/EU.

BALB/c mouse infections were approved by the Institutional Ethics Committee of Animal Experimentation of the National Institute of Medical Sciences and Nutrition Salvador Zubirán in accordance with the guidelines of Mexican national regulations on animal care and experimentation (NOM 062‐ZOO‐1999).

Experiments using armadillos were performed in accordance with USPHS policy on the humane care and use of laboratory animals (67) and USDA Animal and Plant Health Inspection Service guidelines. The Institutional Animal Care and Use Committee reviewed and approved the protocol.
