# Supplementary figures and images for: IL-1R1-Dependent Signals Improve Control of Cytosolic Virulent Mycobacteria In Vivo
Source: mSphere. 2021 May 5;6(3):e00153-21. doi: 10.1128/mSphere.00153-21 (PMC8103984; doi:10.1128/mSphere.00153-21)

Supplemental Fig. S1

Fig. S1

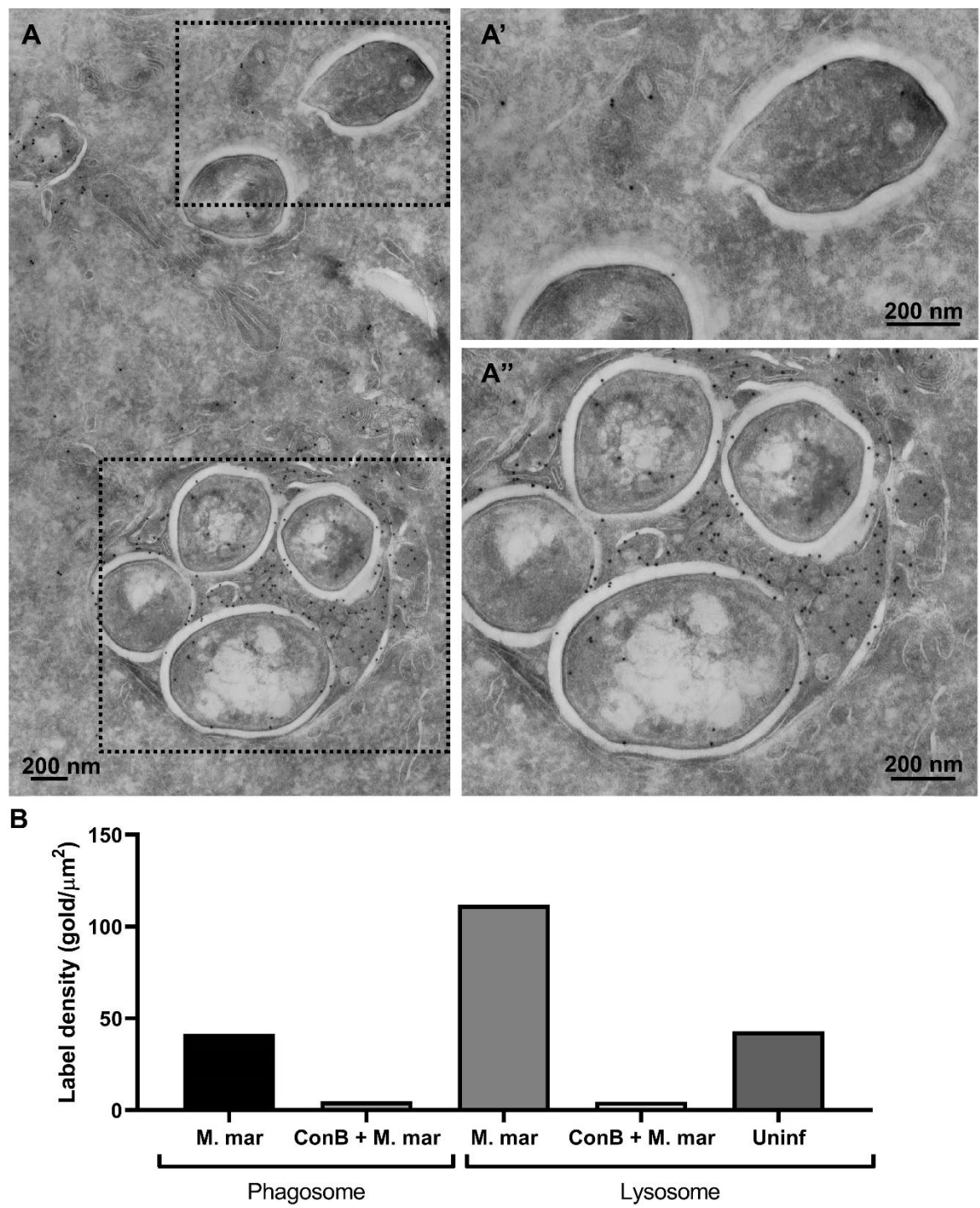

Supplement: FIG S1 [file mSphere.00153-21-sf001.pdf]

# Supplemental Fig. S2

## S Fig. 2

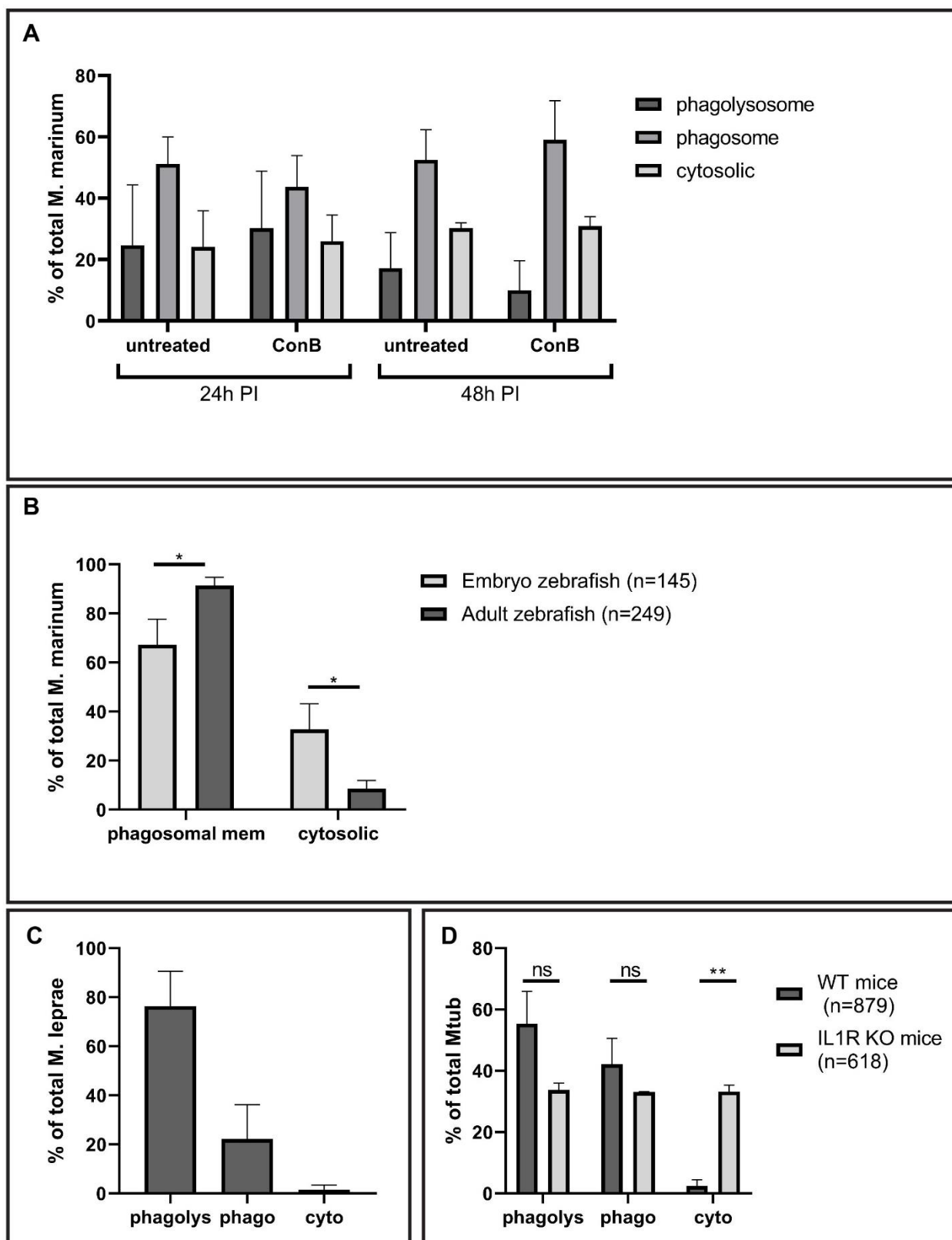

Supplement: FIG S2 [file mSphere.00153-21-sf002.pdf]

Supplemental Fig. S3  
Fig. S3

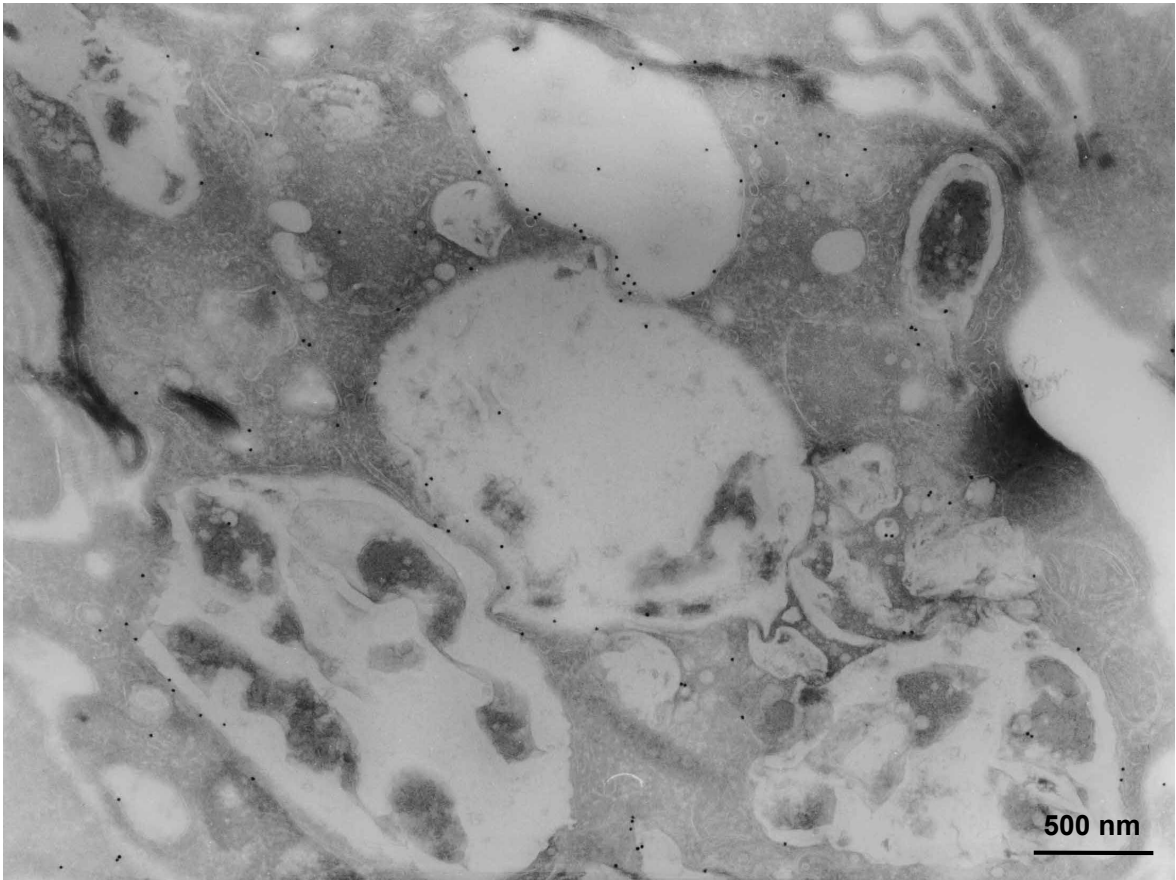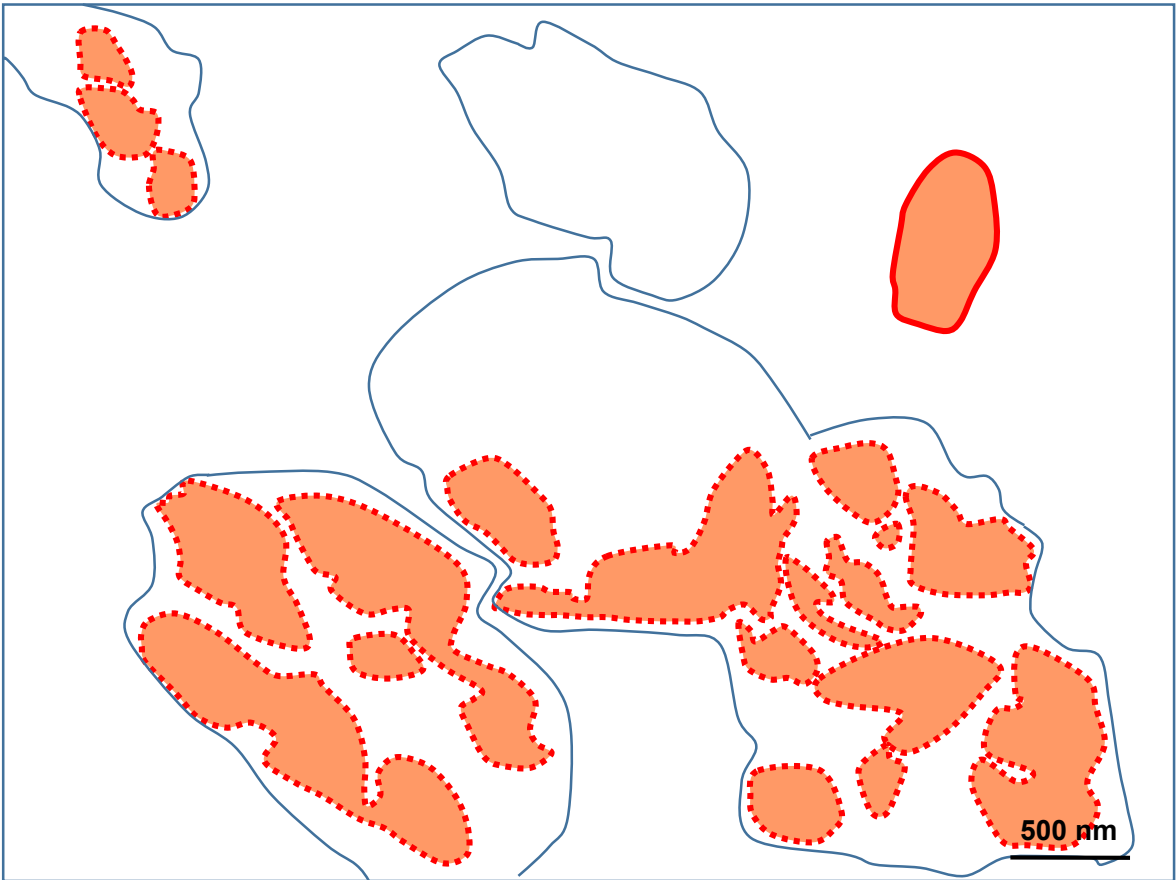

Supplement: FIG S3 [file mSphere.00153-21-sf003.pdf]
