# Supplementary material for: IL-1R1-Dependent Signals Improve Control of Cytosolic Virulent Mycobacteria In Vivo
Source: mSphere. 2021 May 5;6(3):e00153-21. doi: 10.1128/mSphere.00153-21 (PMC8103984; doi:10.1128/mSphere.00153-21)
Supplement: TABLE S1 [file mSphere.00153-21-st001.pdf]

Table S1

Mouse	Mtb strain	Day of infection	% bacteria phago-lysosome	% bacteria phagosome	% bacteria cytosol	n counted bacteria	n mice
BALB/c	H37Rv	D2	38	62	0	47	1
		D7	71	23	6	52	2
		D21	0.4	98	2	247	2
		D45	0	100	0	5	1
		D120	0	97	3	38	2
	1998-1500 Ancient Beijing	D21	0	93	7	31	1
		D45	0	100	0	193	1
		D120	0	100	0	35	2
	2002-0230 Beijing	D120	0	100	0	166	2
SCID	H37Rv	D21	29	54	17	167	1
